# Haloperidol prophylaxis in critically ill patients with a high risk for delirium

**DOI:** 10.1186/cc11933

**Published:** 2013-01-17

**Authors:** Mark van den Boogaard, Lisette Schoonhoven, Theo van Achterberg, Johannes G van der Hoeven, Peter Pickkers

**Affiliations:** 1 Department of Intensive Care Medicine, Radboud University Nijmegen Medical Center, P.O. 9101, 6500HB Nijmegen, The Netherlands; 2 Scientific Institute for Quality of Healthcare, Radboud University Nijmegen Medical Center, P.O. 9101, 6500HB Nijmegen, The Netherlands; 3 Faculty of Health Sciences, University of Southampton, Southampton, UK; 4 Nijmegen Institute for Infection, Inflammation and Immunity (N4i), Radboud University Nijmegen Medical Center, P.O. 9101, 6500HB Nijmegen, The Netherlands

## Abstract

**Introduction:**

Delirium is associated with increased morbidity and mortality. We implemented a delirium prevention policy in intensive care unit (ICU) patients with a high risk of developing delirium, and evaluated if our policy resulted in quality improvement of relevant delirium outcome measures.

**Methods:**

This study was a before/after evaluation of a delirium prevention project using prophylactic treatment with haloperidol. Patients with a predicted risk for delirium of ≥ 50%, or with a history of alcohol abuse or dementia, were identified. According to the prevention protocol these patients received haloperidol 1 mg/8 h. Evaluation was primarily focused on delirium incidence, delirium free days without coma and 28-day mortality. Results of prophylactic treatment were compared with a historical control group and a contemporary group that did not receive haloperidol prophylaxis mainly due to non-compliance to the protocol mostly during the implementation phase.

**Results:**

In 12 months, 177 patients received haloperidol prophylaxis. Except for sepsis, patient characteristics were comparable between the prevention and the historical (*n *= 299) groups. Predicted chance to develop delirium was 75 ± 19% and 73 ± 22%, respectively. Haloperidol prophylaxis resulted in a lower delirium incidence (65% vs. 75%, *P *= 0.01), and more delirium-free-days (median 20 days (IQR 8 to 27) vs. median 13 days (3 to 27), *P *= 0.003) in the intervention group compared to the control group. Cox-regression analysis adjusted for sepsis showed a hazard rate of 0.80 (95% confidence interval 0.66 to 0.98) for 28-day mortality. Beneficial effects of haloperidol appeared most pronounced in the patients with the highest risk for delirium. Furthermore, haloperidol prophylaxis resulted in less ICU re-admissions (11% vs. 18%, *P *= 0.03) and unplanned removal of tubes/lines (12% vs. 19%, *P *= 0.02). Haloperidol was stopped in 12 patients because of QTc-time prolongation (*n *= 9), renal failure (*n *= 1) or suspected neurological side-effects (*n *= 2). No other side-effects were reported. Patients who were not treated during the intervention period (*n *= 59) showed similar results compared to the untreated historical control group.

**Conclusions:**

Our evaluation study suggests that prophylactic treatment with low dose haloperidol in critically ill patients with a high risk for delirium probably has beneficial effects. These results warrant confirmation in a randomized controlled trial.

**Trial registration:**

clinicaltrial.gov Identifier: NCT01187667.

## Introduction

Delirium is a neuropsychiatric disorder characterized by an acute onset of confusion and consciousness alterations that fluctuate during the day [1]. The incidence of delirium in intensive care (ICU) patients is high [2-5], up to 80%, and its occurrence is associated with prolonged duration of mechanical ventilation, increased ICU- and hospital length of stay [3,5], unplanned removal of tubes and catheters [5] and an increased mortality [5,6]. Therefore, preventive treatment for delirium may be beneficial. In non-ICU patients beneficial effects of prophylactic haloperidol in older [7] and surgical ICU patients [8] have been reported. For critically ill patients, data concerning preventive treatment are scarce and inconsistent [7,9-11]. Only prophylactic treatment with the anti-psychotic drug haloperidol seems to have some beneficial effects in ICU patients [12].

In one retrospective cohort study, ICU patients treated with haloperidol appeared to have a lower mortality rate compared to non-treated ICU patients [13]. Another recent study showed that haloperidol prophylaxis in non-cardiac surgical ICU patients had beneficial effects on delirium incidence and delirium free days [12]. Noteworthy, in this study no delirium risk stratification was performed, suggesting that the beneficial effects might be diluted in the whole group of ICU patients and that more pronounced effects may be present in patients with a high risk to develop delirium. Preventive treatment of all ICU patients may not only dilute the potential beneficial effects of haloperidol, but also exposes a substantial number of patients to unnecessary potential risks, for example, the side-effects of haloperidol administration. With the use of a recently developed and validated delirium prediction model for ICU patients [14], patients with a high risk of developing delirium can be identified and targeted preventive treatment becomes possible. In view of the high incidence of delirium, the impact of delirium on outcome and the availability of a delirium prediction model to identify high risk ICU patients, we implemented a delirium prevention protocol in our clinical practice using a low dosage of haloperidol. The aim of this study was to evaluate if our policy resulted in quality improvement of relevant delirium outcome measures.

## Material and methods

### Design and setting

When the decision to implement the delirium prevention protocol was taken, it was prospectively decided to evaluate its effects after one year. A before/after evaluation of a delirium prevention protocol was carried out in our 33-bed intensive care unit (ICU) of the Radboud University Nijmegen Medical Centre, The Netherlands. We wanted to evaluate if our changed delirium policy resulted in improvement of delirium outcome in daily practice. It is recommended to evaluate important changes in daily practice and, according to Dutch law, no approval from an Ethics Committee is warranted when a change in medical policy is evaluated. Nevertheless, we informed our Ethics Committee about the change in policy and the planned analysis upon which it was agreed. This evaluation study was registered in the Clinical trial register (NCT01187667).

### Patients

**Figure 1:**
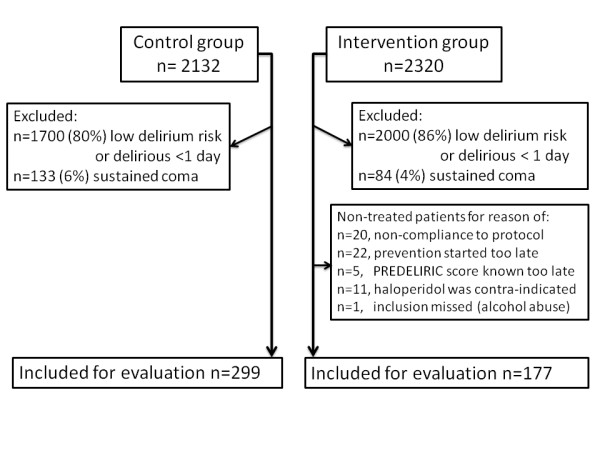
**Flowchart of inclusion of high risk ICU patients**.

All consecutive ICU patients admitted to our intensive care unit between 1 August 2010 and 1 August 2011 were screened for delirium risk and patients with a high risk for delirium (PREdiction DELIRium Intensive Care (PREDELIRIC) score >50% [14], diagnosis of dementia, or alcohol abuse as mentioned in the medical history of the patient) received haloperidol prophylaxis. Following discussion in the ICU staff we defined high risk for delirium as having a PREDELIRIC score [14] of 50% or more. Patients in whom the haloperidol dosage was adjusted or the haloperidol was stopped were for this evaluation allocated to the intervention group. To evaluate the effects of this prevention policy, it was decided beforehand to compare the results with a control group of high risk ICU patients admitted between 1 February 2008 and 1 February 2009 and with a contemporary group of patients who did not receive prophylactic treatment for various reasons (Figure 1) during the intervention period. Also, an additional analysis to determine which delirium risk subgroup ('low, middle, high') within the high risk patients benefitted most from the prophylactic therapy was part of our prospectively agreed analysis plan. Patients in the control group were only treated for delirium and treatment with haloperidol was started as soon as possible after delirium was diagnosed according to our delirium protocol. Patients in the control group and in the intervention were treated at the same ICU location; there were no alterations in environmental conditions during the duration of the study.

### Delirium assessment

All patients were screened by well-trained ICU nurses [15] using the Dutch version of the CAM-ICU [16] at least three times daily, and more often if required. The CAM-ICU screening compliance in the control group was 90.4% and in the intervention group, 94.5%.

To secure the quality of the delirium diagnosis, medical and nursing files of all patients were also screened daily for signs of delirium [17]. When the files contained signs of delirium without a positive CAM-ICU screening or conversely, patients were additionally screened by a delirium expert serving as the 'gold standard', [1] to rule out false negatives and positives. Related to another delirium study [18] we performed these above mentioned additional checks in the historical group as well as in the intervention group.

Patients were excluded when it was impossible to assess the patient for the presence of delirium using the CAM-ICU, that is, because of coma during the entire ICU stay, serious auditory or visual disorders, inability to understand Dutch, severe mental disability or the presence of receptive aphasia. Patients with delirium were divided into three subtypes [19] as measured during the complete delirium period: hyperactive (Richmond agitation sedation score (RASS) [20] +1/+4), hypoactive (RASS 0/-3), and mixed (RASS +4/-3). This last subtype of delirium is characterized by alternating symptoms of hyperactive and hypoactive delirium. Patients were diagnosed with delirium when they had at least one positive CAM-ICU screening during their complete ICU stay. A delirium-and-coma-free day was defined as a negative CAM-ICU screening without RASS -4/-5 during a complete day. Follow-up of all patients was conducted prospectively.

### Delirium prevention

In August 2010 the delirium prevention policy was implemented in daily practice. Patients with an estimated risk of 50% or more determined with the delirium prediction model PRE-DELIRIC [14] and patients with a history of diagnosed dementia or alcohol abuse were considered high risk. The PRE-DELIRIC model is a recently developed and validated delirium prediction model for ICU patients with a high predictive value and consists of 10 predictors, such as age, Acute Physiology and Chronic Health Evaluation II (APACHE-II) score, infection, non-sustained coma, use of sedatives and so on. These predictors are readily available within 24 hours after ICU admission and predict the risk of developing a delirious episode during the complete ICU stay. These high-risk patients received intravenous haloperidol 1 mg/8 h or a lower dose of 0.5 mg/8 h when they were ≥ 80 years, had a body weight <50 kg, had a serum creatinine level >150 μmol/L or had a serum bilirubin level >50 μmol/L. Intravenous haloperidol prophylaxis was started as soon as it was clear when patients had an increased risk, ranging from immediately following ICU admission to 24 hours after ICU admission. If prophylactic therapy was not started within four hours following identification of a high risk patient (24 hours after ICU admission), prophylactic treatment was considered 'too late' and this patient was excluded from the prophylaxis group. In comatose patients, haloperidol was also started; however, if this condition lasted for longer than three days then haloperidol was temporarily stopped until recovery of consciousness. No other anti-psychotics were administered. Patients who developed delirium received therapeutic doses of haloperidol according to the department's protocol.

According to the protocol, prevention was not started when haloperidol was contraindicated in the case of Parkinson's disease, hypokinetic rigid syndrome, Lewy body dementia, prolonged QTc-time of over 500 msec, pregnancy or in patients who were treated with other anti-psychotics.

Since no other changes in the delirium protocol, as well as in other relevant protocols (that is, sedation and pain) were made during the evaluation period, patients in the control group received, except for the haloperidol prophylaxis, the same treatment compared with the delirium prevention group.

### Outcome measures

For this evaluation, primary outcome measures were delirium incidence, number of days alive without delirium and without coma in a period of 28 days, and 28-day mortality. Secondary outcomes were duration of mechanical ventilation, incidence of re-intubation and re-admissions, incidence of unplanned removal of tubes/catheters and ICU- and hospital-length-of-stay. Apart from the analysis of the complete prevention and control group, patients were divided into subgroups based on their predicted chance of developing delirium, to investigate which subgroup of patients benefits most from prophylactic treatment with haloperidol. We also studied outcome in four different admission categories, that is, surgical, medical, trauma and neurological or neurosurgical patients.

Since 2007 delirium has been one of the research topics in our ICU [15], and several delirium related measures have been collected prospectively, with the exception of the PREDELIRIC scores. These were only available for the described control period, as the delirium prediction model was developed in that period.

### Statistical analyses

Demographic characteristics of patients who received haloperidol prophylaxis were compared with non-treated patients. Differences were tested with Student's *t*-test, Mann-Whitney U test or with the Chi-square test, depending on their distribution. Survival analyses with Kaplan-Meier curves as graphical presentation were used. Beforehand it was decided to adjust for variables which differ significantly between the two groups. This decision was based on the fact that the only patients included were those with an increased delirium risk using the PREDELIRIC model consisting of 10 predictors [14]. Therefore, not only the delirium risk should be about equal between the two groups, but also other relevant variables which are part of this prediction model.

To determine the effect of haloperidol on 28-day mortality adjusted for covariates, we used Cox proportional hazard regression analysis with mortality as the dependent variable and baseline characteristics with a *P*-value <0.05 between groups as possible covariates. In case of multicollinearity, determined using Spearman's rho (*P*-value <0.05), the covariate which best associated with the outcome variable mortality was included in the Cox-regression analysis. To examine which patients were most likely to benefit, we equally divided the total group into three risk groups (predicted risk up to 71%, between 71 and 89% and >89%). This distribution was made using the SPSS function 'Visual binning' resulting in an independent optimal distribution in three subgroups. Patient characteristics that differed between groups served as covariates. Statistical significance was defined as a *P-*value <0.05. All data were analyzed using SPSS version 18.0 (SPSS, Chicago, IL, USA).

## Results

During the intervention period (2010 to 2011), 2,320 consecutive ICU patients were screened and 320 patients fulfilled the inclusion criteria for delirium prevention. In the control group (2008 to 2009), 2,132 consecutive ICU patients were screened, of which 432 patients met the inclusion criteria. In the intervention period, a total of 143 patients were excluded and in the control group 133 patients (Figure 1). Overall, 177 patients in the intervention group and 299 patients in the control group were evaluated. Patient and demographic characteristics are shown in Table 1. In the intervention group patients tended to have a slightly lower APACHE-II score, and significantly more patients were admitted with sepsis compared with the control group (*P *= 0.02). Twenty-two (12%) patients in the intervention and 46 (15%) patients in the control group were enrolled because of alcohol abuse or dementia.

**Table 1:** Demographic and patients characteristics

	Control group (*N *= 299)	Intervention group (*N *= 177)	Differences (*P*-value)
Male (n/%)	181 (61%)	115 (65%)	*0.20*
Age	64 ± 14	63 ± 14	*0.64*
APACHE-II score	20 ± 7	19 ± 6	*0.06*
Urgent admission (%)	261 (87%)	152 (86%)	*0.52*
Sedation level (RASS, median (IQR)) - RASS screening compliance (%)	-1 (-3 to 0) 93.3 ± 1.2	-1 (-3 to 0) 94.5 ± 0.9	*0.84*
Haloperidol administering			
- Number of treated patients (%)	225 (75.3%)	177 (100%)	*<0.0001*
- Number of treated days	5 (2 to 12)	5 (3 to 11)*	*0.23*
- Dosage (mg per day) median ((IQR))	6 (3 to 10)	2 (2 to 3)	*<0.0001*
Sepsis (N/%)	64 (21%)	53 (30%)	*0.02*
Admission specialism (N/%):			
- Surgical	75 (25%)	33 (19%)	*0.18*
- Medical	143 (48%)	106 (60%)	*0.12*
- Trauma	32 (11%)	18 (10%)	*0.27*
- Neurology/neurosurgical	49 (16%)	20 (11%)	*0.36*
PRE-DELIRIC score	73 ± 22	75 ± 19	*0.50*
Other risk			
- Alcohol abuse	41 (14%)	20 (11%)	*0.37*
- Dementia	5 (2%)	2 (1%)	

* Including prophylactic days of treatment. Data are presented as mean ± standard deviation unless mentioned otherwise.

### Primary outcomes

**Figure 2:**
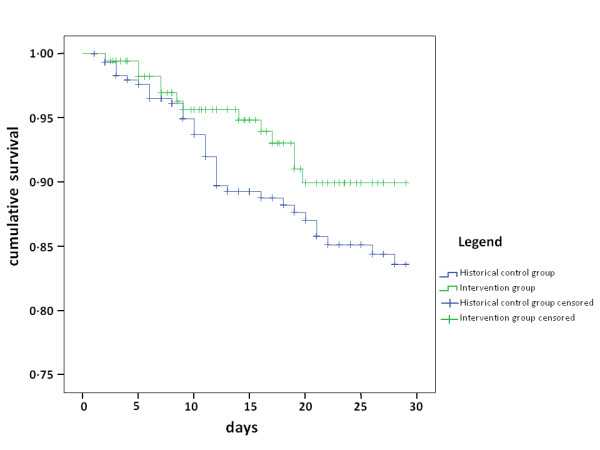
**Kaplan-Meier survival plot of 28-day survival**.

The predicted chance of developing delirium in the intervention and control group was 75 ± 19% and 73 ± 22%, respectively (*P *= 0.50). The actual delirium incidence was 65% in the intervention group, compared with 75% in the control group (*P *= 0.01) (Table 2). The number of delirium free days was significantly higher in the intervention group (median 20 days (interquartile range 8 to 27) versus median 13 days (3 to 27) (*P *= 0.003)). Cox proportional hazard regression analysis was performed with sepsis as a covariate. Prophylactic treatment with haloperidol resulted in a relative 28-day mortality reduction of 20% (hazard rate 0.80; 95% CI 0.66 to 0.98). Figure 2 shows the 28-day Kaplan-Meier survival curve of both groups. Since multicollinearity was determined between sepsis and the APACHE-II score (borderline significant difference between the two groups) we used only sepsis as a covariate.

**Table 2:** Differences between control group and complete intervention group

	Control group (*N *= 299)	Intervention group (*N *= 177)	Differences (*P*-value)
*Predicted delirium chance *	*73 ± 22*	*75 ± 19*	***0.50***
Observed delirium incidence (n,%)	225 (75%)	115 (65%)	*0.01*
Non-delirium:	74 (25%)	62 (35%)	*0.38*
Delirium subtype:			
- Hyperactive	20 (7%)	6 (3%)	
- Hypoactive	81 (27%)	33 (19%)	
- Mixed	124 (41%)	76 (43%)	
Number of delirium free days without coma in 28 days	13 (3 to 27)	20 (8 to 27)	*0.003*
Re-intubation (%)	25 (8%)	15 (9%)	*0.51*
Duration mechanical ventilation in hrs.	118 (39 to 250)	90 (36 to 229)	*0.24*
Unplanned removal tubes/lines (%)	58 (19%)	21 (12%)	*0.02*
- Tube	8 (3%)	4 (2%)	
- Gastric tube	26 (9%)	14 (8%)	
- CVC/arterial line	24 (8%)	1 (<1%)	
- Other	0 (0%)	2 (1%)	
Re-admission	55 (18%)	20 (11%)	*0.03*
LOS-ICU	7 (3 to 13)	6 (3 to 12)	*0.65*
LOS-in hospital	21 (12 to 41)	20 (11 to 31)	*0.16*
28-day mortality	36 (12%)	13 (7.3%)	0.03*

Data are presented as median, interquartile range (IQR), unless mentioned otherwise. * Cox proportional hazard regression analysis adjusted for sepsis and cohort (hazard rate 0.80; 95% CI 0.66 to 0.98.

### Secondary outcomes

No significant differences were found between groups in duration of mechanical ventilation, ICU- and hospital-length-of-stay, and incidence of re-intubation. Patients who received prophylaxis were less likely to remove their tubes and catheters (*P *= 0.02) and were less likely to be re-admitted to the ICU (*P *= 0.03) (Table 2).

### Risk groups and admission categories

To examine which patients benefit most from the prophylactic therapy with haloperidol, the total group was equally divided into three groups based on their predicted risk. Patients with the highest risk appear to benefit most from the prophylactic treatment with haloperidol (Table 3), although no statistically significant difference between groups was reached for all end points.

**Table 3:** Differences between control group and complete intervention group divided in three delirium risk groups

Predicted chance <71%	Control group (*N *= 110)	Intervention group (*N *= 69)	Differences (*P*-value)
Predicted chance	50 ± 19	55 ± 16	*0.08*
Age	63 ± 13	63 ± 14	*0.88*
APACHE-II score	17 ± 5	16 ± 5	*0.12*
Sepsis (%)	11 (10%)	18 (26%)	*0.005*
Observed delirium incidence	55 (50%)	30 (44%)	*0.27*
28 days delirium free without coma	26 (10 to 28)	26 (13 to 28)	*0.17*
28-day mortality	13 (12%)	6 (9%)	0.34*
Re-intubation (%)	5 (5%)	6 (9%)	*0.25*
Duration mechanical ventilation in hrs.	42 (14 to 150)	63 (15 to 168)	*0.43*
Unplanned removal tubes/lines (%)	14 (13%)	7 (10%)	*0.41*
- Tube	1 (1%)	1 (2%)	
- Gastric tube	2 (2%)	4 (6%)	
- CVC/arterial line	11(10%)	1 (2%)	
- Other	0 (0%)	1 (<1%)	
Re-admission	18 (16%)	4 (6%)	*0.03*
LOS-ICU	3 (2 to 8)	4 (2 to 8)	*0.32*
LOS-in hospital	17 (9 to 31)	16 (8 to 27)	*0.48*

***Predicted chance 71 to 89% ***	*(N = 111)*	*(N = 60)*	*Differences *

Predicted chance	81 ± 5	80 ± 5	*0.66*
Age	64 ± 14	61 ± 15	*0.14*
APACHE-II score	20 ± 6	20 ± 7	*0.94*
Sepsis (%)	31 (28%)	24 (40%)	*0.08*
Observed delirium incidence	94 (85%)	44 (73%)	*0.06*
28 days delirium free without coma	11 (3 to 22)	20 (7 to 27)	*0.02*
28-day mortality	13 (12%)	5 (8%)	*0.93*
Re-intubation (%)	11 (11%)	6 (10%)	*0.56*
Duration mechanical ventilation in hrs.	124 (55 to 278)	133 (50 to 281)	*0.76*
Unplanned removal tubes/lines (%)	22 (20%)	8 (13%)	*0.20*
- Tube	4 (4%)	2 (3%)	
- Gastric tube	12 (11%)	6 (10%)	
- CVC/arterial line	6 (5%)	0 (0%)	
Re-admission	27 (24%)	12 (20%)	*0.33*
LOS-ICU	8 (3 to 15)	8 (4 to 17)	*0.57*
LOS-in hospital	23 (13 to 43)	26 (16 to 41)	*0.99*

***Predicted chance >89% ***	*(N = 78)*	*(N = 48)*	*Differences *

Predicted chance	95 ± 3	95 ± 3	*0.95*
Age	62 ± 16	65 ± 12	*0.34*
APACHE-II score	24 ± 8	21 ± 6	*0.05*
Sepsis (%)	22 (28%)	11(23%)	*0.33*
Observed delirium incidence	76 (97%)	41 (85%)	*0.06*
28 days delirium free without coma	4 (0 to 14)	13 (6 to 21)	*0.002*
28-day mortality	10 (13%)	2 (4%)	*0.07^†^*
Re-intubation (%)	9 (12%)	3 (6%)	*0.25*
Duration mechanical ventilation in hrs.	185 (112 to 353)	94 (62 to 266)	*0.02*
Unplanned removal tubes/lines (%)	22 (28%)	6 (13%)	*0.04*
- Tube	3 (4%)	1 (2%)	
- Gastric tube	12 (15%)	3 (6%)	
- CVC/arterial line	7 (9%)	0 (0%)	
- Other	0 (0%)	2 (4%)	
Re-admission	10 (13%)	4 (8%)	*0.32*
LOS-ICU	11 (7 to 18)	6 (4 to 15)	*0.03*
LOS-in hospital	30 (14 to 56)	20 (15 to 31)	*0.07*

Data are presented as median, interquartile range (IQR), unless mentioned otherwise. * Cox proportional hazard regression analysis with sepsis as covariate. *^† ^*Cox proportional hazard regression analysis with APACHE-II score as covariate.

Results of prophylactic haloperidol treatment for the different admission categories are shown in Appendix A (Additional file 1). The beneficial effects of prophylactic treatment were comparable between patient groups. Medical patients appeared to benefit most from prophylactic haloperidol treatment.

### Non-treated patients during the implementation period

During the implementation period, a total of 59 patients did not receive prophylaxis with haloperidol, mostly due to non-compliance to the new protocol in the early phase of implementation. There were no demographic differences between the control group and this non-treated group (Table 4). However, there were significantly more patients with sepsis in the non-treated intervention group compared with the control group. Also, there was a small, non-significant difference in case-mix among the three groups.

**Table 4:** Non-treated patients in the intervention group compared with treated and control group

	Control group (*N *= 299)	Intervention group non-treated (*N *= 59)	Intervention group treated (*N *= 177)
PRE-DELIRIC score (mean ± sd)	73 ± 22	77 ± 17	75 ± 19
Other risk			
- Alcohol abuse	41 (14%)	4 (7%)	20 (11%)
- Dementia	5 (2%)	0	2 (1%)
Age (mean ±)	64 ± 14	62 ± 15	63 ± 14
Urgent admission (%)	261 (87%)	52 (88%)	152 (86%)
APACHE-II score	20 ± 7	20 ± 6	19 ± 6
Sepsis (%)	64 (21%)^b^	16 (28%)^a^	53 (30%)^a^
Gender (M/%)	181 (61%)	35 (59%)	115 (65%)
Admission specialism (N/%):			
- Surgical	75 (25%)	11 (19%)	33 (19%)
- Medical	143 (48%)	30 (51%)	106 (60%)
- Trauma	32 (11%)	5 (9%)	18 (10%)
- Neurology/neurosurgical	49 (16%)	13 (22%)^c^	20 (11%)

Delirium incidence	225 (75%)^b^	53 (90%)^a-b^	115 (65%)
28 days delirium free without coma	13 (3 to 27)^b^	14 (1 to 22)^b^	20 (8 to 27)
28-day mortality	36 (12%)^b^	7 (12%)	13 (7%)
Re-intubation (%)	25 (8%)	8 (14%)	15 (9%)
Duration mechanical ventilation in hrs.	118 (39 to 250)	103 (54 to 251)	90 (36 to 229)
Unplanned removal tubes/lines (%)	58 (19%)^b^	13 (22%)^b^	21 (12%)
Re-admission	55 (18%)^b^	13 (22%)^b^	20 (11%)
LOS-ICU	7 (3 to 13)	7 (4 to 14)	6 (3 to 12)
LOS-in hospital	21 (12 to 41)	27 (13 to 48)^b^	20 (11 to 31)

Data are presented as median, interquartile range (IQR), unless mentioned otherwise. ^a ^Statistically significantly different (*P *<0.05) compared with control group. ^b ^Statistically significantly different (*P *<0.05) compared with treated intervention group. ^c ^Borderline significant difference between non-treated and treated intervention group (*P *= 0.057).

The incidence of delirium, unplanned removal of tubes and re-admission rate was significantly higher and the number of delirium free days was significantly lower in the non-treated group compared with the treated intervention group. In addition, the delirium incidence in the non-treated intervention group was also significantly higher compared with the treated intervention group (Table 4).

### Haloperidol treatment

All patients were examined daily for signs of rigidity, restlessness, Parkinsonism, QTc-time measurement and level of sedation. In 14 out of 177 (8%) patients, adjustments in dosage were made because of possible side effects, with drowsiness as the most frequently mentioned reason (6%). Haloperidol was stopped in 12 (7%) patients because of prolonged QTc-time (*n *= 9, all in patients treated with mild hypothermia), signs of Parkinsonism (*n *= 1), renal failure (*n *= 1) and in one patient malignant neuroleptic syndrome was suspected, but later not confirmed. Importantly, none of the nine patients developed any tachyarrhythmia during the prolonged QTc-time period.

## Discussion

This before/after evaluation study suggests that prophylactic treatment with haloperidol in ICU patients with a high risk for delirium results in a lower delirium incidence, more delirium free days and a reduction in mortality. In addition, the evaluation of our delirium prevention policy shows that patients that received prophylactic haloperidol were less likely to remove their tubes or catheters or to be readmitted to the ICU, also illustrating the beneficial effects of prophylactic therapy with haloperidol. Importantly, only a few side-effects of low dose haloperidol were reported of which none were severe. The results of our evaluation study need to be confirmed in a study with a more powerful design. Although our study design is not optimal, our results are in accordance with previous studies in which prophylactic treatment with haloperidol was successfully used in older and surgical patients [8] and recently in a group of non-cardiac surgical ICU patients [12].

While prophylactic therapy for delirium is sparsely studied in critically ill patients, more data are available concerning the treatment of delirium in hospitalized patients. Haloperidol is recommended as the first choice drug for delirium treatment [1,21-23]. Several studies showed that use of other anti-delirium drugs than haloperidol [24-26] do not further improve patient outcome, but could even worsen it [27]. However, one small study showed that the use of other anti-delirium drugs in combination with haloperidol improved delirium outcome [28]. Some of these randomized trials demonstrated less severe or shorter duration of delirium in the treatment groups, but were underpowered to detect an effect on length of stay or mortality [24-26]. In an observational study, a lower mortality rate in ICU patients was observed in ICU patients treated with haloperidol compared to those that were not treated [13]. In addition, observational data also suggest that early treatment of delirium results in a lower mortality rate compared with delayed treatment [29]. How haloperidol decreases mortality is unknown. Knowing the energy consuming nature of delirium, it appears plausible that patients suffering from delirium become exhausted, eventually resulting in respiratory insufficiency. The higher incidence of re-intubations, ICU readmissions and diversion of the Kaplan-Meier curves from Day 10 are in accordance with this assumption. These data indicate that the effectiveness of early treatment, or possibly even better prophylaxis, may be superior compared to treatment of delirium. However, prophylactic treatment of all ICU patients inherently results in a number of patients who are unnecessarily exposed to the side-effects of haloperidol, which may harm the patient [30]. In the study of Wang *et al. *[12], all non-cardiac surgical ICU patients were included irrespective of their level of risk, indicating that overtreatment occurred. Furthermore, this may have diluted the prophylactic effect of haloperidol in the patients with a high chance of developing delirium. Therefore, there is a need for a delirium prediction model for ICU patients, which identifies the patients with a high risk of developing delirium. In the present study, we used our delirium prediction model with a high predictive value [14]. Importantly, our data suggest that the higher the predicted risk, the more effective prophylaxis with haloperidol is. Also, it seems that medical patients benefit more than surgical patients, which may be due to the higher delirium risk in these patients, or the differences in the underlying mechanism of delirium.

Several limitations need to be addressed. Most importantly, we performed a before/after evaluation study instead of a more powerful and controlled design, such as a randomized controlled trial. Nevertheless, the fact that a better outcome was observed in patients that received prophylactic haloperidol also compared to non-prophylactically treated patients during the intervention period, could indicate that the results were not relevantly confounded by a time-dependent bias. In addition, we chose relevant end-points known to be related to delirium. In view of the congruent effects of prophylactic treatment with haloperidol on several end points, the plausibility of our findings is further supported. Importantly, during the complete study there were no important changes in medical policy concerning analgesia as well as for sedation, illustrated by similar RASS scores in the two groups. Therefore, it appears unlikely that time-dependent bias played an important role in our study. However, we appreciate that this observational retrospective cohort study, including a contemporary cohort, is not an optimal design to draw firm conclusions. Therefore, a randomized controlled trial is necessary to confirm our conclusions.

Second, we did not use the 'gold standard', the DSM-IV criteria [1], to diagnose delirium in daily ICU practice, but we used the CAM-ICU of which it was recently determined the performance is somewhat lower [31,32] than in the original studies of Ely *et al. *[31-34]. Importantly, in view of the fluctuating nature of delirium, all patients were screened three times daily and more often if needed. When delirium was not detected with the CAM-ICU, but delirium was suspected based on medical and nursing reports, patients were additionally screened by a delirium expert according to the DSM-IV criteria. Furthermore, in a previous study we determined that the inter-rater reliability of the CAM-ICU screenings by our ICU nurses was high [15]. We, therefore, assume that only a few patients may be misdiagnosed, which is likely equally distributed between the study periods.

Third, potential side-effects of haloperidol were only observed when spontaneously reported and mild extrapyramidal side-effects may have been missed, although daily thorough physical examination of all patients is the usual care in our ICU. Regarding QTc-time, this was measured daily using an electrocardiogram (ECG). In nine patients haloperidol was stopped because of prolonged QTc-time, which was probably due to the immediate start of haloperidol in the post-cardiopulmonary resuscitation phase after ICU admission combined with mild therapeutic hypothermia [35] in all nine patients. Moreover, QTc-time was only marginally prolonged and none of these patients developed ventricular arrhythmia, as reported in some case reports [36-39], or other tachyarrhythmias. Furthermore, in several patients, haloperidol dose was adjusted for reasons of drowsiness or a possible sedative effect. Importantly, all these patients were delirious and these symptoms may also represent manifestations of delirium [1,19]. The low incidence of side effects is in accordance with previous studies [7,8,40,41].

Fourth, the choice of the haloperidol dose likely influences the treatment effect. Our dosage was lower than the 5 mg/day that was used in surgery patients [8], which also resulted in a reduction of the delirium incidence. In view of the few reported side-effects of haloperidol in our study and the other ICU study [12], and the still relatively high delirium incidence rate in ICU patients that received prophylactic treatment, a higher prophylactic dosage should be considered in future research.

Lastly, patients who were not preventively treated according to our delirium prevention protocol, mostly due to non-compliance, served as an additional control group. Although this group showed similar patient characteristics as the historical control group and the prophylactic treated intervention group, the outcome measures in this group were comparable with the historical control group. This supports the beneficial effects of prophylactic treatment with haloperidol.

## Conclusions

The use of the delirium prevention protocol seems to result in improvement of several delirium outcome measures. Prophylactic treatment with low dose haloperidol in critically ill patients with a high risk of delirium likely appears to exert relevant beneficial effects. With the encouraging results of the recent and present studies, we feel that a randomized prospective intervention study in ICU patients with a high risk for delirium using prophylactic haloperidol should be conducted. It should be considered, given the few side-effects of a low dose haloperidol, to also investigate the effect of a higher prophylactic dosage of haloperidol.

## Key messages

• Identifying high risk with a delirium prediction model (PREDELIRIC) facilitates targeted delirium prevention.

• Low dosage of prophylactic haloperidol in ICU patients with a high risk for delirium is associated with a lower incidence of delirium, more delirium-free days and a lower mortality.

• Also, complications related to delirium (for example, unintended removal of lines and tubes) were less frequent in patients that received prophylactic treatment with haloperidol.

• Medical patients and those with the highest delirium risk appear to benefit the most.

## Abbreviations

APACHE-II: Acute Physiology and Chronic Health Evaluation-II; CAM-ICU: confusion-assessment method, intensive care unit; DSM-IV: Diagnostic and Statistical Manual of Mental Disorders-IV; ECG: electrocardiogram; PREDELIRIC: PREdiction DELIRium Intensive Care; RASS: Richmond Agitation Sedation Score;

## Competing interests

All authors declare that they have no conflicts of interest.

## Authors' contributions

MvdB carried out the evaluation, the statistics and drafted the manuscript. LS and PP were involved in and supervised the evaluation and helped to draft the manuscript. JvdH and TvA supervised the evaluation and corrected the manuscript. All authors read and approved the final manuscript.

## Supplementary Material

Additional file 1 **Results of the prophylactic haloperidol treatment in the different admission categories**. Results of the prophylactic haloperidol treatment in the admission category: surgical patients, medical patients, trauma patients and neurology/neurosurgical patients compared with the control group. Click here for file
