# Supplementary material for: Haloperidol prophylaxis in critically ill patients with a high risk for delirium
Source: Crit Care. 2013 Jan 17;17(1):R9. doi: 10.1186/cc11933 (PMC4056261; doi:10.1186/cc11933)
Supplement: Additional file 1 — Results of the prophylactic haloperidol treatment in the different admission categories. Results of the prophylactic haloperidol treatment in the admission category: surgical patients, medical patients, trauma patients and neurology/neurosurgical patients compared with the control group. [file cc11933-S1.DOC]

**Appendix A.** Supplementary table online available

**Results of the prophylactic haloperidol treatment in the different admission categories**

| ***Surgical patients*** | *Control group*  *(N=75)* | *Intervention group*  *(N=33)* | *Differences*  *(P-value)* |
| --- | --- | --- | --- |
| Predicted chance (mean, SD) | 62±25 | 62±24 | *0.68* |
| Observed delirium incidence | 47 (63%) | 18 (55%) | *0.28* |
| 28 days delirium free without coma | 13 [3-28] | 22 [10-28] | *0.05* |
| 28-day mortality | 6 (8%) | 1 (3%) | *0.31* |
| Re-intubation (%) | 7 (9%) | 3 (9%) | *0.64* |
| Duration mechanical ventilation in hrs. | 57 [16-181] | 20 [6-170] | *0.21* |
| Unplanned removal tubes/lines (%) | 12 (16%) | 5 (15%) | *0.58* |
| Re-admission | 15 (20%) | 7 (21%) | *0.54* |
| LOS-ICU | 8 [4-15] | 3 [2-9] | *0.55* |
| LOS-in hospital | 23 [12-45] | 20 [11-37] | *0.92* |
| ***Medical patients*** | *(N=143)* | *(N=106)* | *Differences* |
| Predicted chance (mean, SD) | 75±20 | 77±17 | *0.97* |
| Observed delirium incidence | 116 (81%) | 69 (65%) | *0.003* |
| 28 days delirium free without coma | 11 [3-22] | 20 [7-27] | *0.04* |
| 28-day mortality | 20 (14%) | 9 (9%) | *0.13* |
| Re-intubation (%) | 11 (11%) | 9 (9%) | *0.56* |
| Duration mechanical ventilation in hrs. | 153 [72-330] | 120 [63-260] | *0.17* |
| Unplanned removal tubes/lines (%) | 26 (18%) | 9 (9%) | *0.02* |
| Re-admission | 30 (21%) | 11 (10%) | *0.02* |
| LOS-ICU | 8 [3-15] | 7 [4-14] | *0.36* |
| LOS-in hospital | 23 [13-43] | 20 [11-34] | *0.06* |
| ***Trauma patients*** | *(N=32)* | *(N=18)* | *Differences* |
| Predicted chance (mean, SD) | 76±16 | 71±18 | *0.35* |
| Observed delirium incidence | 22 (69%) | 12 (67%) | *0.56* |
| 28 days delirium free without coma | 14 [0-27] | 20 [11-27] | *0.59* |
| 28-day mortality | 1 (3%) | 0 (0%) | *0.64* |
| Re-intubation (%) | 3 (9%) | 2 (11%) | *0.61* |
| Duration mechanical ventilation in hrs. | 80 [17-284] | 62 [19-261] | *0.02* |
| Unplanned removal tubes/lines (%) | 9 (28%) | 5 (28%) | *0.62* |
| Re-admission | 1 (3%) | 0 (0%) | *0.64* |
| LOS-ICU | 8 [3-14] | 5 [4-16] | *0.86* |
| LOS-in hospital | 22 [14-40] | 23 [15-28] | *0.77* |
| ***Neurology/neurosurgical patients*** | *(N=49)* | *(N=20)* | *Differences* |
| Predicted chance (mean, SD) | 82±18 | 87±16 | *0.26* |
| Observed delirium incidence | 40 (82%) | 16 (80%) | *0.56* |
| 28 days delirium free without coma | 14 [4-26] | 18 [15-27] | *0.14* |
| 28-day mortality | 9 (19%) | 3 (15%) | *0.52* |
| Re-intubation (%) | 7 (14%) | 1 (5%) | *0.28* |
| Duration mechanical ventilation in hrs. | 112 [36-220] | 71 [36-175] | *0.43* |
| Unplanned removal tubes/lines (%) | 11 (22%) | 2 (10%) | *0.22* |
| Re-admission | 9 (18%) | 2 (10%) | *0.32* |
| LOS-ICU | 6 [3-14] | 6 [4-10] | *0.89* |
| LOS-in hospital | 20 [9-37] | 19 [15-25] | *0.70* |

Data are presented as median, interquartile range [IQR], unless mentioned otherwise
